# Health-related quality of life in patients with newly diagnosed multiple myeloma ineligible for stem cell transplantation: results from the randomized phase III ALCYONE trial

**DOI:** 10.1186/s12885-021-08325-2

**Published:** 2021-06-02

**Authors:** Stefan Knop, Maria-Victoria Mateos, Meletios A. Dimopoulos, Kenshi Suzuki, Andrzej Jakubowiak, Chantal Doyen, Paulo Lucio, Zsolt Nagy, Ganna Usenko, Ludek Pour, Mark Cook, Sebastian Grosicki, Andre Crepaldi, Anna Marina Liberati, Philip Campbell, Tatiana Shelekhova, Sung-Soo Yoon, Genadi Losava, Tomoaki Fujisaki, Mamta Garg, Jianping Wang, Susan Wroblewski, Anupa Kudva, Katharine S. Gries, John Fastenau, Jesus San-Miguel, Michele Cavo

**Affiliations:** 1 Department of Haematology and Oncology, Würzburg University Medical Center, Oberdürrbacher Straße 6, 97080 Würzburg, Germany; 2 University Hospital of Salamanca–Instituto de Investigación Biomédica de Salamanca, Salamanca, Spain; 3 National and Kapodistrian University of Athens, Athens, Greece; 4 Japanese Red Cross Medical Center, Tokyo, Japan; 5 University of Chicago Medical Center, Chicago, IL USA; 6 Université Catholique of Louvain, CHU UCL Namur, Yvoir, Belgium; 7 Champalimaud Centre for the Unknown, Lisbon, Portugal; 8 Semmelweis University, Budapest, Hungary; 9 Dnipropetrovsk City Multidisciplinary Clinical Hospital No. 4, Dnipropetrovsk, Ukraine; 10 University Hospital Brno, Brno, Czech Republic; 11 University Hospitals Birmingham NHS Trust, Birmingham, UK; 12 Silesian Medical University, Katowice, Poland; 13 Clinica de Tratamento E, Cuiaba, Brazil; 14 Azienda Ospedaliera “Santa Maria”, Terni, Italy; 15 Andrew Love Cancer Centre, Geelong, Australia; 16 Clinic of Professional Pathology, Saratov, Russia; 17 Department of Internal Medicine, Seoul National University College of Medicine, Seoul, Republic of Korea; 18 LTD “Medinvent” Institute of Health, Tbilisi, Georgia; 19 Matsuyama Red Cross Hospital, Matsuyama, Japan; 20 Leicester Royal Infirmary, Leicester, UK; 21 Janssen Research and Development, Raritan, NJ USA; 22 Janssen Research & Development, LLC, Spring House, PA USA; 23 Clínica Universidad de Navarra–Centro de Investigación Médica Aplicada, Instituto de Investigación Sanitaria de Navarra, Centro de Investigación Biomédica en Red de Cáncer, Pamplona, Spain; 24 Institute of Hematology, Department of Experimental, Diagnostic and Specialty Medicine, University of Bologna, Bologna, Italy

## Abstract

**Background:**

In the phase III ALCYONE trial, daratumumab plus bortezomib/melphalan/prednisone (D-VMP) significantly improved overall response rate and progression-free status compared with VMP alone in transplant-ineligible patients with newly diagnosed multiple myeloma (NDMM). Here, we present patient-reported outcomes (PROs) from ALCYONE.

**Methods:**

The European Organisation for Research and Treatment of Cancer Quality of Life Questionnaire Core 30-item (EORTC QLQ-C30) and EuroQol 5-dimensional descriptive system (EQ-5D-5L) questionnaire were administered at baseline, every 3 months (year 1) and every 6 months (until progression). Treatment effects were assessed using a repeated-measures, mixed-effects model.

**Results:**

Compliance with PRO assessments was comparable at baseline (> 90%) and throughout study (> 76%) for both treatment groups. Improvements from baseline were observed in both groups for EORTC QLQ-C30 Global Health Status (GHS), most functional scales, symptom scales and EQ-5D-5L visual analog scale (VAS). Between-group differences were significant for GHS (*p* = 0.0240) and VAS (*p* = 0.0160) at month 3. Improvements in pain were clinically meaningful in both groups at all assessment time points. Cognitive function declined in both groups, but the magnitude of the decline was not clinically meaningful.

**Conclusions:**

Patients with transplant-ineligible NDMM demonstrated early and continuous improvements in health-related quality of life, including improvements in functioning and symptoms, following treatment with D-VMP or VMP.

**Trial registration:**

ClinicalTrials.gov identifier NCT02195479, registered September 21, 2014

**Supplementary Information:**

The online version contains supplementary material available at 10.1186/s12885-021-08325-2.

## Background

Treatment approaches for newly diagnosed multiple myeloma (NDMM) are chosen based on the patient’s fitness; those considered fit usually receive induction, high-dose chemotherapy and autologous stem cell transplant (ASCT) as standard of care [1]. In patients ineligible for ASCT, treatment with bortezomib, melphalan and prednisone (VMP) or lenalidomide plus low-dose dexamethasone is recommended [1]. Older patients and those who are transplant ineligible have significantly shorter relative survival than younger, fitter, transplant-eligible patients [2, 3]. In addition to age, factors such as frailty, performance status and comorbidities are important determinants of ASCT eligibility and treatment selection in the frontline setting [4–6].

MM can profoundly impact patients’ daily lives, imposing both physical (i.e. fatigue, mobility, pain and physical activity) and emotional (i.e. distress, anxiety, depression and effects on relationships) burdens [7, 8]. Maintaining health-related quality of life (HRQoL) during treatment is an important goal in MM, with a particular focus on understanding the long-term impact of disease and treatment on patients [9]. However, reports on HRQoL in the ASCT-ineligible population (in particular) are limited.

In May 2018, the US Food and Drug Administration approved daratumumab, an anti-CD38 humanized monoclonal antibody, for use in combination with VMP (D-VMP) in patients with NDMM who are ineligible for ASCT. This approval was based on the results of the multicenter, open-label, phase III ALCYONE trial (NCT02195479), which demonstrated significantly higher response rates, higher rates of minimal residual disease negativity, and lower risk of disease progression or death in patients who received D-VMP compared with those receiving VMP alone. Prespecified subgroup analyses showed the superiority of D-VMP over VMP in patients 75 years of age or older (29.9% of patients in the study) and those with poor prognosis [10]. Rates of grade 3/4 hematologic events, including neutropenia, thrombocytopenia and anemia, were higher in the D-VMP group than in the VMP group [10].

Here, we present analyses from the ALCYONE clinical trial evaluating the treatment effect of D-VMP on patient-reported outcomes (PROs).

## Methods

### Study design and patients

Details of the multicenter, randomized, open-label, active-controlled, parallel group ALCYONE trial have been previously published [10]. Cycle length for D-VMP and VMP was 6 weeks (cycles 1–9) and 4 weeks (cycle 10+), respectively. Eligible patients were randomized 1:1 to D-VMP (VMP [see below] plus intravenous daratumumab 16 mg/kg [once weekly in cycle 1, every 3 weeks in cycles 2–9 and every 4 weeks thereafter until disease progression or unacceptable toxicity]) or VMP (subcutaneous bortezomib 1.3 mg/m^2^ [cycle 1: twice weekly; cycles 2–9: 4 doses/cycle], melphalan 9 mg/m^2^ [days 1–4] and prednisone 60 mg/m^2^ [days 1–4]).

The study was conducted at 162 sites in 25 countries. Each study site’s local independent ethics committee or institutional review board approved the study protocol. This study was conducted in accordance with the ethical principles that have their origin in the Declaration of Helsinki and the International Conference on Harmonisation Good Clinical Practice guidelines and adhered to CONSORT guidelines. All patients provided written informed consent.

### PROs

PROs (a secondary objective of the ALCYONE trial) were assessed by the European Organisation for Research and Treatment of Cancer Quality of Life Questionnaire Core 30-item (EORTC QLQ-C30) [11] and the EuroQol 5-dimensional descriptive system (EQ-5D-5L) [12]. The EORTC QLQ-C30 v3 is a validated, cancer-specific instrument that contains 30 items resulting in five functional scales (physical, role, emotional, cognitive and social functioning), one Global Health Status (GHS) scale, three symptom scales (fatigue, nausea and vomiting, and pain) and six single items (dyspnea, insomnia, appetite loss, constipation, diarrhea and financial difficulties) [11]. Higher scores represent greater GHS, better functioning and worse symptoms, respectively. The EQ-5D-5L, a generic measure of health status, assesses five domains including mobility, self-care, usual activities, pain/discomfort and anxiety/depression plus a visual analog scale (VAS) rating of “health today” [12].

PRO responses were collected using an electronic tablet device prior to any other study-related activities, at baseline (before randomization), every 3 months during the treatment phase and then every 6 months until disease progression. All patients were educated on the use of the electronic tablet. Interim results are presented for the first 36 months of treatment.

### Statistical methods

The primary analysis population was the intent-to-treat (ITT) population (all randomized patients); the PRO data set was the ITT population of patients with a baseline and > 1 postbaseline PRO assessment. No imputation of missing data or adjustments for multiplicity were made. A sensitivity analysis was conducted using a pattern-mixture model.

PRO data were summarized using descriptive statistics, including number, mean, standard deviation, median, and minimum and maximum value by treatment group. Compliance was calculated at baseline and for each postbaseline PRO assessment visit as a percentage, with the number of PRO assessments received as the numerator and the number of PRO assessments expected at that time point (a clinical prediction of how many patients will be on treatment) as the denominator.

We assessed treatment differences using a repeated-measures, mixed-effects model with a missing-at-random data assumption. The model included the baseline PRO score, treatment group, time, treatment by time interaction and the stratification factors as fixed effects and subject as a random effect. A 2-sided 5% significance level was used to descriptively compare values for the exploratory PRO endpoints, which are derived from scale scores.

The proportion of patients achieving minimally important differences (MIDs) in each PRO instrument scale score, which indicate clinically meaningful changes, was summarized with odds ratios and 95% confidence intervals (CIs). Although there is no universal MID [13], there are multiple published MID thresholds ranging from 5 to 10 [14–17]. Here, MID thresholds to explore individual patient-level change were defined a priori as 10 points for the EORTC QLQ-C30 scale scores [18] and ≥ 7 points for EQ-5D-5L VAS [19].

We conducted exploratory subgroup analyses to determine if there were differences in EORTC QLQ-C30 GHS, functional and symptom scale scores by age, and Eastern Cooperative Oncology Group (ECOG) performance status. Exploratory analysis of time to worsening using survival curves and hazard ratios (HRs) by depth of clinical response and minimal residual disease (MRD) status were estimated for EORTC QLQ-C30 scores.

## Results

### Patients

Baseline patient demographics and population characteristics were similar between groups (D-VMP: *n* = 350; VMP: *n* = 356) (Table 1). Mean age was 71 years, there were approximately the same number of male and female patients, and approximately half of patients had a baseline ECOG performance status of 1. Baseline EORTC QLQ-C30 scores were similar between treatment arms for all functional and symptom scales (Table 1).

**Table 1:** Baseline characteristics, EORTC QLQ-C30 scores and EQ-5D-5L scores (ITT population)

Characteristic	D-VMP (***n*** = 350)	VMP (***n*** = 356)
Age, years		
Mean (SD)	71.3 (6.66)	71.5 (5.82)
< 65, *n* (%)	36 (10.3)	24 (6.7)
65 to < 75, *n* (%)	210 (60.0)	225 (63.2)
≥ 75, *n* (%)	104 (29.7)	107 (30.1)
Male, *n* (%)	160 (45.7)	167 (46.9)
Race, *n* (%)		
White	297 (84.9)	304 (85.4)
Black or African American	3 (0.9)	3 (0.8)
Asian	47 (13.4)	45 (12.6)
Other, Unknown or Not reported	3 (0.9)	4 (1.1)
ECOG performance status, *n* (%)		
0	78 (22.3)	99 (27.8)
1	182 (52.0)	173 (48.6)
2	90 (25.7)	84 (23.6)
EORTC QLQ-C30 GHS score, mean (SD)		
GHS	50.74 (20.996)	52.40 (22.691)
EORTC QLQ-C30 functional scores, mean (SD)		
Physical functioning	59.96 (26.756)	63.65 (25.701)
Role functioning	57.54 (34.250)	61.06 (33.192)
Emotional functioning	69.70 (24.735)	71.10 (22.304)
Cognitive functioning	80.49 (21.998)	83.38 (19.948)
Social functioning	70.41 (28.468)	70.64 (28.651)
EORTC QLQ-C30 symptom scores, mean (SD)		
Pain	46.10 (33.130)	43.12 (31.429)
Fatigue	42.48 (25.661)	41.05 (25.798)
Nausea/vomiting	5.38 (12.425)	5.45 (13.566)
Dyspnea	18.35 (24.930)	19.37 (24.742)
Insomnia	29.54 (30.044)	27.93 (31.135)
Appetite loss	22.26 (28.116)	21.81 (26.623)
Constipation	23.00 (31.517)	18.86 (27.404)
Diarrhea	5.91 (17.015)	5.40 (15.270)
Financial difficulties	18.88 (26.534)	17.33 (24.743)
EQ-5D-5L scores, mean (SD)	(*n* = 316)	(*n* = 325)
VAS	57.72 (20.254)	60.32 (20.556)

*SD* standard deviation

Higher EORTC QLQ-C30 scores represent greater GHS, better functioning and worse symptoms. Higher EQ-5D-5L VAS score represents better health

Compliance rates with PRO measures were high and similar across treatment groups. At baseline, 90.6 and 90.3% of patients subsequently assigned to the D-VMP group and 91.9 and 91.3% of patients randomized to the VMP group completed the EORTC QLQ-C30 and the EQ-5D-5L, respectively (supplementary Fig. 1). Compliance rates remained high (> 76%) throughout the study. The number of PRO assessments received was higher in the D-VMP group than in the VMP group, which is consistent with the greater numbers of patients staying on treatment with D-VMP compared with VMP. The PRO data sets for the D-VMP group were also larger owing to the longer treatment duration of these patients.

### Treatment effect on EORTC QLQ-C30 scores

#### ITT population

**Fig. 1:**
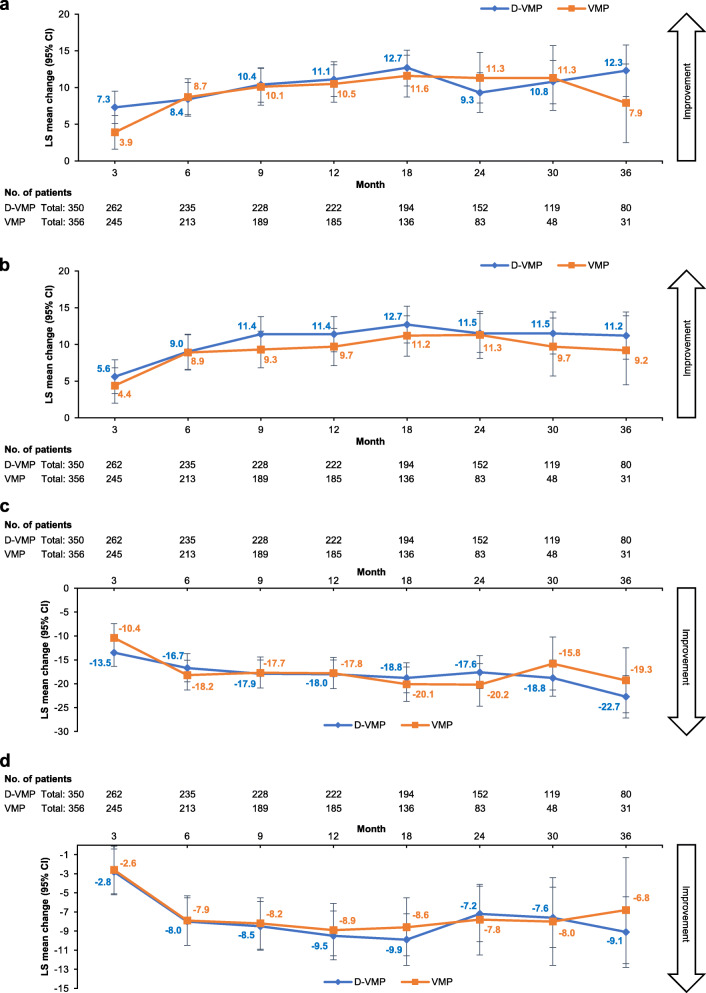
LS mean change from baseline in EORTC QLQ-C30. **a** – GHS. **b** – Physical functioning. **c** – Pain. **d** – Fatigue up to 36 months (ITT population)

Using a mixed-effects model with repeated measures, the least squares (LS) mean change in GHS score from baseline was 7.3 in the D-VMP group and 3.9 in the VMP group at 3 months (difference 3.4, *p* = 0.0240). Between-group differences were not significant at other assessment time points, but point estimates more often favored the D-VMP group than the VMP group. The LS mean change from baseline was clinically meaningful (i.e. ≥10 points) at months 9, 12, 18 and 30 for both treatment groups, as well as at month 24 in the VMP group and month 36 in the D-VMP group (Fig. 1a).

LS mean changes from baseline were not significantly different between treatment groups for the functional scales of the EORTC QLQ-C30. Point estimates favored the D-VMP group at all assessment time points for physical functioning (Fig. 1b) and most time points for role functioning, cognitive functioning and social functioning (supplementary Fig. 2a, c, d). The direction of the point estimates for between-group differences in change in emotional functioning fluctuated depending on the assessment time point, favoring D-VMP at months 6, 12, 30 and 36 and favoring VMP at months 3, 9, 18 and 24 (supplementary Fig. 2b). LS mean change from baseline in physical functioning scores was more often clinically meaningful in the D-VMP group than in the VMP group (6 vs 2 assessment time points). Clinically meaningful changes in role functioning scores were observed in the D-VMP group at all time points after month 3 and in the VMP group at all time points between month 3 and month 30. LS mean changes in emotional functioning scores were clinically meaningful in both treatment groups at all assessment time points after month 3. Scores for cognitive functioning declined from baseline in both groups, but the LS mean change from baseline was not clinically meaningful in either group at any assessment time point.

There were no significant between-group differences in LS mean change from baseline in scores for pain (Fig. 1c), fatigue (Fig. 1d) or nausea and vomiting (supplementary Fig. 2e). Point estimates generally favored D-VMP for these symptoms. Improvements in pain scores were clinically meaningful at all assessment time points for both treatment groups, but were not meaningful for either group at any time point for fatigue or nausea and vomiting. Although overall usage of concomitant medications was similar between groups (97.7 and 96.9% of patients in the D-VMP and VMP groups, respectively), greater proportions of patients in the D-VMP group than in the VMP group used analgesics (70.8% vs 57.9%) and anti-inflammatory agents (25.7% vs 14.4%).

**Fig. 2:**
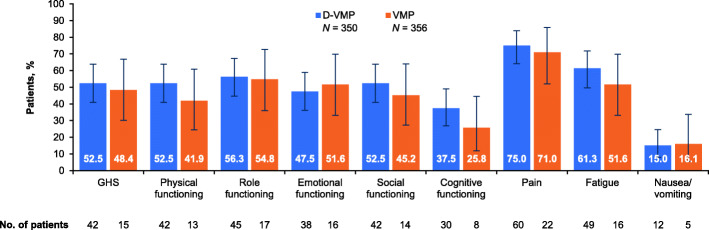
Percentage of patients reporting clinically meaningful improvements in EORTC QLQ-C30 functional and symptom scales at 36 months. Clinically meaningful improvement defined as a ≥ 10-point improvement from baseline score

The proportions of patients with a clinically meaningful change (i.e. ≥10 points) in EORTC QLQ-C30 scores at month 36 are shown in Fig. 2. Differences between treatment groups were not statistically significant, but were numerically greater in the D-VMP group for all scales. The greatest proportion of patients experienced clinically meaningful changes in pain scores, with 75% of patients in the D-VMP group and 71% of patients in the VMP group reporting a mean change ≥10 points.

#### Subgroup analyses

In a subgroup analysis of EORTC QLQ-C30 GHS, physical functioning, pain and fatigue scores by age and ECOG performance status, similar patterns of increasing improvement in HRQoL were observed in all subgroups, including patients 75 years of age or older and those with poorer overall baseline functional status (ECOG performance status ≥2), with no significant difference between the D-VMP and VMP groups after 3 months (Table 2). Improvements were observed in all subgroups, but were generally greater in the younger (< 75 years) vs older (≥75 years) patients and in those with ECOG performance status of 2 vs those with ECOG performance status of 0 or 1.

**Table 2:** Mean change from baseline in EORTC QLQ-C30 GHS, physical functioning, pain and fatigue and EQ-5D-5L VAS scores up to 36 months by age and ECOG performance status at baseline

	Months									
	3		6		12		24		36	
LS mean change from baseline (95% CI)	VMP	D-VMP	VMP	D-VMP	VMP	D-VMP	VMP	D-VMP	VMP	D-VMP
EORTC QLQ-C30 GHS										
< 75 years	(*n* = 178)	(*n* = 193)	(*n* = 156)	(*n* = 171)	(*n* = 143)	(*n* = 163)	(*n* = 64)	(*n* = 115)	(*n* = 21)	(*n* = 68)
	6.5	10.7	12.4	11.9	13.7	15.2	14.7	14	12.1	16.4
	(4.1–8.9)	(8.4–13)	(9.9–15)	(9.4–14.3)	(11.1–16.4)	(12.7–17.7)	(11–18.4)	(11.1–16.9)	(6–18.2)	(12.8–19.9)
≥ 75 years	(*n* = 65)	(*n* = 69)	(*n* = 56)	(*n* = 64)	(*n* = 42)	(*n* = 59)	(*n* = 18)	(*n* = 37)	(*n* = 10)	(*n* = 12)
	4.2	5.5	6.2	6.2	9.8	7.5	9.9	3.6	4.4	8.5
	(−0.3 to 8.7)	(1–10)	(1.3–11.1)	(1.6–10.8)	(4.2–15.3)	(2.8–12.3)	(2.1–17.8)	(−2.2 to 9.4)	(−6.1 to 15)	(−1.1 to 18.2)
ECOG performance status 0	(*n* = 68)	(*n* = 56)	(*n* = 57)	(*n* = 46)	(*n* = 51)	(*n* = 51)	(*n* = 25)	(*n* = 34)	(*n* = 11)	(*n* = 16)
	−0.7	8.5	4.8	7.5	1.7	8.5	6	7.4	1.3	10.3
	(−4.9 to 3.5)	(3.8–13.1)	(0.3–9.3)	(2.5–12.5)	(−3 to 6.4)	(3.7–13.3)	(−0.5 to 12.5)	(1.7–13.1)	(−8.2 to 10.8)	(2.3–18.3)
ECOG performance status 1	(*n* = 123)	(*n* = 138)	(*n* = 108)	(*n* = 126)	(*n* = 95)	(*n* = 115)	(*n* = 38)	(*n* = 73)	(*n* = 13)	(*n* = 43)
	5.6	7.8	9.5	8.7	12.2	11.6	11	7.4	9.3	11.8
	(2.7–8.6)	(5–10.6)	(6.4–12.7)	(5.8–11.6)	(9–15.5)	(8.6–14.6)	(6.1–15.8)	(3.8–11)	(1.4–17.1)	(7.3–16.3)
ECOG performance status 2	(*n* = 54)	(*n* = 68)	(*n* = 48)	(*n* = 63)	(*n* = 39)	(*n* = 56)	(*n* = 20)	(*n* = 45)	(*n* = 7)	(*n* = 21)
	11.6	14.7	18	17.9	24.2	21.5	24.2	23.3	19.3	24.7
	(6.9–16.3)	(10.5–18.9)	(13.1–23)	(13.6–22.2)	(18.8–29.6)	(17–26)	(17.2–31.3)	(18.4–28.2)	(8.1–30.6)	(18–31.4)
EORTC QLQ-C30 physical functioning										
< 75 years	(*n* = 178)	(*n* = 193)	(*n* = 156)	(*n* = 171)	(*n* = 143)	(*n* = 163)	(*n* = 64)	(*n* = 115)	(*n* = 21)	(*n* = 68)
	8.4	10	12.7	14.1	14.9	17.2	16.8	16.9	14.6	16.9
	(6.1–10.7)	(7.7–12.2)	(10.3–15.1)	(11.8–16.4)	(12.5–17.4)	(14.8–19.5)	(13.5–20)	(14.3–19.5)	(9.5–19.8)	(13.7–20)
≥ 75 years	(*n* = 65)	(*n* = 69)	(*n* = 56)	(*n* = 64)	(*n* = 42)	(*n* = 59)	(*n* = 18)	(*n* = 37)	(*n* = 10)	(*n* = 12)
	2.9	3	8	4.4	4.1	4.9	5.1	5.9	3.9	3.5
	(−2 to 7.9)	(−1.9 to 7.8)	(2.8–13.1)	(−0.5 to 9.4)	(−1.6 to 9.8)	(− 0.2 to 9.9)	(− 2.4 to 12.6)	(0.1–11.7)	(− 5.8 to 13.6)	(− 5.5 to 12.5)
ECOG performance status 0	(*n* = 68)	(*n* = 56)	(*n* = 57)	(*n* = 46)	(*n* = 51)	(*n* = 51)	(*n* = 25)	(*n* = 34)	(*n* = 11)	(*n* = 16)
	−1.2	2.8	3.1	6.1	2.3	4.6	1.3	2	−0.6	2.4
	(−4.5 to 2.1)	(−0.8 to 6.5)	(−0.4 to 6.6)	(2.8–10.4)	(−1.3 to 5.9)	(0.9–8.4)	(−3.3 to 5.9)	(−2.3 to 6.2)	(−7.1 to 5.8)	(−3.2 to 8)
ECOG performance status 1	(*n* = 123)	(*n* = 138)	(*n* = 108)	(*n* = 126)	(*n* = 95)	(*n* = 115)	(*n* = 38)	(*n* = 73)	(*n* = 13)	(*n* = 43)
	5.2	4.6	9.5	7.3	8.7	11	13.6	10.1	9.9	8.5
	(2.1–8.3)	(1.7–7.6)	(6.3–12.7)	(4.3–10.3)	(5.3–12)	(7.9–14.1)	(9–18.2)	(6.6–13.7)	(2.8–16.9)	(4.2–12.7)
ECOG performance status 2	(*n* = 54)	(*n* = 68)	(*n* = 48)	(*n* = 63)	(*n* = 39)	(*n* = 56)	(*n* = 20)	(*n* = 45)	(*n* = 7)	(*n* = 21)
	16.8	21.4	22.4	26.5	29.3	29.4	27.1	33.4	27.9	35.3
	(11.3–22.3)	(16.5–26.2)	(16.7–28.1)	(21.6–31.5)	(23.3–35.3)	(24.3–34.6)	(19.8–34.4)	(28–38.8)	(17.1–38.8)	(28.4–42.1)
EORTC QLQ-C30 pain*										
< 75 years	(*n* = 178)	(*n* = 193)	(*n* = 156)	(*n* = 171)	(*n* = 143)	(*n* = 163)	(*n* = 64)	(*n* = 115)	(*n* = 21)	(*n* = 68)
	−12.9	−16.4	−22.1	−20.2	−21.8	−21.7	−24.8	− 23.3	−26.4	− 27
	(− 16 to − 9.9)	(− 19.3 to − 13.4)	(−25.3 to − 18.8)	(−23.3 to − 17.1)	(− 25.2 to − 18.5)	(− 24.8 to − 18.5)	(− 29.5 to − 20.1)	(− 26.9 to − 19.7)	(−34.1 to − 18.8)	(− 31.5 to − 22.6)
≥ 75 years	(*n* = 65)	(*n* = 69)	(*n* = 56)	(*n* = 64)	(*n* = 42)	(*n* = 59)	(*n* = 18)	(*n* = 37)	(*n* = 10)	(*n* = 12)
	−9.6	− 12.4	− 14.1	− 13.7	−13.2	− 14.5	− 14.3	−8.1	− 8.8	− 17.9
	(− 15.4 to − 3.8)	(− 18.2 to − 6.7)	(− 20.3 to − 7.8)	(− 19.6 to − 7.8)	(− 20.2 to − 6.1)	(− 20.6 to − 8.4)	(− 24.4 to − 4.3)	(− 15.5 to − 0.7)	(− 22.3 to 4.7)	(− 30.3 to − 5.6)
ECOG performance status 0	(*n* = 68)	(*n* = 56)	(*n* = 57)	(*n* = 46)	(*n* = 51)	(*n* = 51)	(*n* = 25)	(*n* = 34)	(*n* = 11)	(*n* = 16)
	− 1.6	− 0.7	− 10	− 7.3	− 6.5	− 5.2	− 9.5	−8.5	− 11.7	− 12.8
	(− 6.3 to 3)	(− 5.9 to 4.4)	(− 15.1 to − 5)	(− 12.9 to − 1.7)	(− 11.7 to − 1.2)	(− 10.6 to 0.1)	(− 16.7 to − 2.3)	(− 14.8 to − 2.1)	(− 22.2 to − 1.2)	(− 21.6 to − 3.9)
ECOG performance status 1	(*n* = 123)	(*n* = 138)	(*n* = 108)	(*n* = 126)	(*n* = 95)	(*n* = 115)	(*n* = 38)	(*n* = 73)	(*n* = 13)	(*n* = 43)
	− 9.6	− 15.5	− 18.8	− 16.6	− 16.3	− 18.6	−19	− 16.2	− 14.8	− 23
	(− 13.6 to − 5.6)	(− 19.2 to − 11.7)	(− 23 to − 14.6)	(− 20.5 to − 12.7)	(− 20.7 to − 11.9)	(− 22.6 to − 14.5)	(− 25.4 to − 12.5)	(− 21 to − 11.3)	(− 25.3 to − 4.4)	(− 29.1 to − 17)
ECOG performance status 2	(*n* = 54)	(*n* = 68)	(*n* = 48)	(*n* = 63)	(*n* = 39)	(*n* = 56)	(*n* = 20)	(*n* = 45)	(*n* = 7)	(*n* = 21)
	− 26.6	− 29	− 31.4	− 33.5	− 40	− 36.4	− 40.8	− 36.4	− 41.1	− 39.9
	(−32.7 to − 20.4)	(− 34.5 to − 23.5)	(− 37.9 to − 24.9)	(− 39.2 to − 27.9)	(− 47.1 to − 33)	(− 42.3 to − 30.5)	(− 50 to − 31.6)	(− 42.8 to − 29.9)	(− 55.8 to − 26.5)	(− 48.7 to − 31.1)
EORTC QLQ-C30 fatigue^a^										
< 75 years	(*n* = 178)	(*n* = 193)	(*n* = 156)	(*n* = 171)	(*n* = 143)	(*n* = 163)	(*n* = 64)	(*n* = 115)	(*n* = 21)	(*n* = 68)
	−7.3	− 7.5	−12.9	− 13.5	−14.1	− 14.9	− 13.6	−13.2	− 12.3	−14.7
	(− 9.9 to − 4.7)	(− 10.1 to − 5)	(− 15.7 to − 10.2)	(− 16.1 to − 10.9)	(− 16.9 to − 11.2)	(− 17.6 to − 12.2)	(− 17.5 to − 9.7)	(− 16.2 to − 10.1)	(− 18.6 to − 5.9)	(− 18.5 to − 11)
≥ 75 years	(*n* = 65)	(*n* = 69)	(*n* = 56)	(*n* = 64)	(*n* = 42)	(*n* = 59)	(*n* = 18)	(*n* = 37)	(*n* = 10)	(*n* = 12)
	0	0	−4.3	− 3.1	− 4.7	− 4.8	−1.5	− 0.6	− 2.2	− 2.9
	(− 5 to 5)	(− 4.8 to 4.9)	(− 9.6 to 0.9)	(− 8.1 to 1.9)	(− 10.6 to 1.3)	(− 10 to 0.4)	(− 9.7 to 6.7)	(− 6.7 to 5.6)	(− 13 to 8.6)	(− 12.8 to 7)
ECOG performance status 0	(*n* = 68)	(*n* = 56)	(*n* = 57)	(*n* = 46)	(*n* = 51)	(*n* = 51)	(*n* = 25)	(*n* = 34)	(*n* = 11)	(*n* = 16)
	5.5	−2.1	−2.9	− 6.3	− 0.7	− 6	1	− 3.8	−1.2	− 0.9
	(1.2–9.9)	(− 6.9 to 2.7)	(−7.5 to 1.7)	(− 11.4 to − 1.2)	(− 5.4 to 4)	(− 11 to − 1.1)	(− 5.1 to 7.1)	(−9.4 to 1.8)	(− 9.7 to 7.3)	(−8.3 to 6.5)
ECOG performance status 1	(*n* = 123)	(*n* = 138)	(*n* = 108)	(*n* = 126)	(*n* = 95)	(*n* = 115)	(*n* = 38)	(*n* = 73)	(*n* = 13)	(*n* = 43)
	− 5.6	− 5.3	− 11.4	−8.7	− 11.1	− 10.8	− 8.9	−8.3	− 7.4	− 11.1
	(− 8.9 to − 2.2)	(− 8.5 to − 2.2)	(− 14.9 to − 7.9)	(− 12 to − 5.5)	(− 14.8 to − 7.4)	(− 14.2 to − 7.5)	(− 14.2 to − 3.7)	(− 12.3 to − 4.4)	(− 15.9 to 1)	(− 16 to − 6.2)
ECOG performance status 2	(*n* = 54)	(*n* = 68)	(*n* = 48)	(*n* = 63)	(*n* = 39)	(*n* = 56)	(*n* = 20)	(*n* = 45)	(*n* = 7)	(*n* = 21)
	− 15	− 10.2	− 15.4	− 20	− 23.7	− 21.7	− 25	− 19.5	− 19.5	− 23.7
	(− 20.3 to − 9.7)	(− 14.9 to − 5.5)	(− 20.9 to − 9.8)	(− 24.8 to − 15.1)	(− 29.7 to − 17.8)	(− 26.8 to − 16.7)	(− 32.8 to − 17.3)	(− 25 to − 14.1)	(− 31.7 to − 7.4)	(− 31.1 to − 16.4)
EQ-5D-5L VAS										
< 75 years	(*n* = 178)	(*n* = 193)	(*n* = 156)	(*n* = 171)	(*n* = 143)	(*n* = 163)	(*n* = 64)	(*n* = 115)	(*n* = 21)	(*n* = 68)
	6.8	9.7	9.7	10.9	12.8	12.5	15.3	13.2	13.9	15.6
	(4.7–8.8)	(7.7–11.6)	(7.5–11.8)	(8.8–12.9)	(10.6–15)	(10.4–14.6)	(12.2–18.3)	(10.8–15.6)	(9–18.9)	(12.7–18.6)
≥ 75 years	(*n* = 65)	(*n* = 69)	(*n* = 56)	(*n* = 64)	(*n* = 42)	(*n* = 59)	(*n* = 18)	(*n* = 37)	(*n* = 10)	(*n* = 12)
	2	5.7	4.5	6	8.1	2.9	3.4	5.1	3.8	2.6
	(−1.9 to 5.9)	(1.9–9.5)	(0.3–8.6)	(2.1–9.9)	(3.5–12.8)	(−1.1 to 6.9)	(−3.1 to 10)	(0.3–9.8)	(−4.7 to 12.3)	(− 5.1 to 10.4)
ECOG performance status 0	(*n* = 68)	(*n* = 56)	(*n* = 57)	(*n* = 46)	(*n* = 51)	(*n* = 51)	(*n* = 25)	(*n* = 34)	(*n* = 11)	(*n* = 16)
	− 0.5	6.8	2.6	7.9	4.4	4	5.4	6	1.4	4.9
	(−3.7 to 2.7)	(3.3–10.3)	(−0.9 to 6)	(4.1–11.8)	(0.9–8)	(0.4–7.7)	(0.6–10.3)	(1.7–10.3)	(−5.5 to 8.2)	(−0.9 to 10.8)
ECOG performance status 1	(*n* = 123)	(*n* = 138)	(*n* = 108)	(*n* = 126)	(*n* = 95)	(*n* = 115)	(*n* = 38)	(*n* = 73)	(*n* = 13)	(*n* = 43)
	5.1	6.8	6.8	6.7	10.7	8.5	10.4	7.4	11.8	10.1
	(2.4–7.7)	(4.3–9.3)	(4–9.6)	(4.1–9.3)	(7.8–13.6)	(5.9–11.2)	(6.2–14.6)	(4.3–10.6)	(5–18.5)	(6.2–14.1)
ECOG performance status 2	(*n* = 54)	(*n* = 68)	(*n* = 48)	(*n* = 63)	(*n* = 39)	(*n* = 56)	(*n* = 20)	(*n* = 45)	(*n* = 7)	(*n* = 21)
	11.4	15.2	16.3	18.5	20	19.2	22.8	23.3	20.3	26.1
	(7.6–15.3)	(11.7–18.7)	(12.2–20.4)	(15–22.1)	(15.6–24.4)	(15.5–22.9)	(17.1–28.5)	(19.3–27.3)	(11.3–29.2)	(20.7–31.5)

^a^For pain and fatigue, a negative change equates to greater symptom improvement

Time to worsening of GHS, function and symptoms was generally longer with greater depth of clinical response (Table 3). Time to worsening was significantly longer for patients with a complete response and significantly shorter for patients with stable disease, both compared with patients with very good partial response/partial response on the EORTC QLQ-C30 GHS (hazard ratio = 0.72 and 1.75, respectively). Similarly, patients who reached MRD-negative status had significantly improved outcomes compared with MRD-positive patients on EORTC QLQ-C30 GHS and pain (hazard ratio = 0.70 and 0.60, respectively; *p* < 0.05).

**Table 3:** HRs for comparison of time to worsening of EORTC QLQ-C30 scale scores by depth of response and MRD status for pooled treatment arms

EORTC QLQ-C30 scales	Clinical response				MRD^**−**^ vs MRD^**+**^	
	(s)CR vs VGPR/PR		SD vs VGPR/PR			
	HR (95% CI)	***p*** value	HR (95% CI)	***p*** value	HR (95% CI)	***p*** value
GHS	0.72 (0.54–0.96)	0.025	1.75 (1.16–2.66)	0.008	0.70 (0.50–0.99)	0.042
Physical functioning	0.88 (0.66–1.17)	0.365	1.60 (0.05–2.45)	0.031	0.79 (0.57–1.10)	0.168
Role functioning	0.93 (0.72–1.20)	0.578	1.55 (1.05–2.27)	0.027	0.84 (0.62–1.12)	0.235
Emotional functioning	0.79 (0.57–1.07)	0.120	1.87 (1.21–2.88)	0.005	0.93 (0.66–1.31)	0.680
Cognitive functioning	0.81 (0.64–1.02)	0.075	1.28 (0.88–1.85)	0.200	1.02 (0.78–1.32)	0.910
Social functioning	0.87 (0.67–1.13)	0.290	1.50 (1.01–2.23)	0.044	0.89 (0.66–1.21)	0.453
Pain	0.72 (0.56–0.94)	0.017	1.22 (0.81–1.86)	0.340	0.60 (0.43–0.83)	0.002
Fatigue	1.01 (0.88–1.29)	0.950	1.44 (0.99–2.10)	0.060	0.98 (0.74–1.29)	0.023
Nausea/vomiting	1.05 (0.80–1.39)	0.733	1.38 (0.88–2.16)	0.158	0.92 (0.67–1.27)	0.610
Dyspnea	0.64 (0.48–0.84)	0.002	1.42 (0.94–2.15)	0.096	0.71 (0.51–0.98)	0.037
Insomnia	0.80 (0.62–1.04)	0.099	1.26 (0.84–1.90)	0.265	0.83 (0.61–1.12)	0.215
Appetite loss	1.05 (0.80–1.39)	0.716	1.67 (1.10–2.55)	0.017	0.96 (0.71–1.31)	0.596
Constipation	0.80 (0.61–1.06)	1.127	1.30 (0.84–2.02)	0.233	0.92 (0.67–1.26)	0.596
Diarrhea	1.03 (0.77–1.38)	0.832	1.39 (0.87–2.24)	0.172	0.89 (0.63–1.24)	0.475

*PR* partial response; *(s)CR* (stringent) complete response; *SD* stable disease; *VGPR* very good partial response

Worsening of EORTC QLQ-C30 GHS defined as a ≥ 10-point decrease and worsening of EORTC QLQ-C30 pain and fatigue defined as a ≥ 10-point increase

### Treatment effect on EQ-5D-5L scores

#### ITT population

**Fig. 3:**
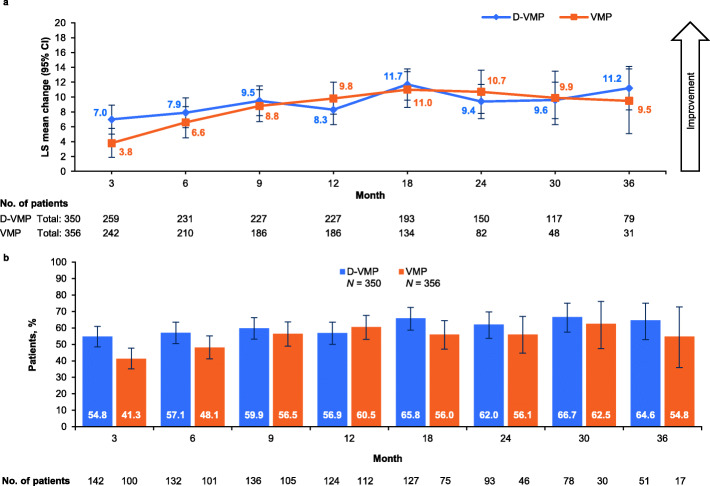
EQ-5D-5L VAS. **a** – LS mean change from baseline up to 36 months. **b** – Percentage of patients reporting clinically meaningful improvements up to 36 months (ITT population)

At month 3, the LS mean difference from baseline in EQ-5D-5L VAS score was 7 in the D-VMP group and 3.8 in the VMP group (difference 3.1, *p* = 0.0160). Between-group differences were not significant at other assessment time points. Point estimates favored the D-VMP group at months 6, 9, 18 and 36 and favored the VMP group at months 12, 24 and 30 (Fig. 3a). The LS mean change was clinically meaningful in both groups at month 18, in the VMP group at month 24 and in the D-VMP group at month 36. The proportion of patients with a clinically meaningful improvement in VAS score was significantly greater in the D-VMP group at month 3 (54.8% vs 41.3%, odds ratio 1.72, *p* = 0.0025); between-group differences were not significant at other time points (Fig. 3b). The proportion of patients with clinically meaningful improvement in VAS score was numerically greater in the D-VMP group at all assessment time points except month 12.

#### Subgroup analyses

Similar to the subgroup analysis for EORTC QLQ-C30, increasing improvement over time was observed for EQ-5D-5L VAS scores in subgroups by age and ECOG performance status, with no significant difference between treatment groups after 3 months (Table 2). Improvements were greater in younger patients and those with an ECOG performance status of 2.

## Discussion

MM is incurable and was responsible for 1.1% of all cancer deaths worldwide in 2018 [20], and patients with MM experience high levels of pain, fatigue and mood disturbances [21]. MM treatments are often associated with demanding administration and monitoring schedules, as well as adverse events. As a result, the burden of MM on patients’ HRQoL is substantial, and PROs should be an important consideration for evaluating new treatment strategies in these patients. This is particularly relevant for the subpopulation of patients with NDMM who are ineligible for transplant, as this group is typically older and often has comorbidities, including impaired renal and hepatic function, that may limit therapeutic options and/or increase susceptibility to adverse effects. Currently, data on HRQoL in this patient subpopulation are limited.

The results presented here provide clear evidence of the HRQoL benefits of D-VMP and VMP in patients with NDMM who are not eligible for transplant. These findings are consistent with a systematic review by Nielsen et al. [22], which reported clinically relevant improvement in HRQoL following treatment in this population. Our study is the first to examine HRQoL of patients treated with D-VMP, and the robustness of the results was supported by a sensitivity analysis using a pattern-mixture model.

Clinically meaningful improvements in GHS, function and symptoms were maintained in this patient population to at least 36 months, which corresponds to the median overall survival of the control group in the Myeloma Trialists’ Collaborative Group meta-analysis of 24 randomized MM trials [23], and is likely among the longest durations of follow-up reported in the first-line treatment of transplant-ineligible patients with MM. Baseline health status and burden of disease measured using the EORTC QLQ-C30 GHS, functional scales and symptom scales were worse for patients with NDMM compared with a population-based random sample of adults without cancer in Germany [24]. Nevertheless, post-treatment scores improved to a level approaching those of a noncancer population [25]. Improvements in cognitive functioning were lower than those reported for the other functional scales, but this is likely attributable to a ceiling effect, as the mean baseline scores for the cognitive functioning scale were the highest of the functional scales, leaving little additional room for improvement, especially in patients on active treatment. The improvements in pain and fatigue observed with both D-VMP and VMP may be particularly noteworthy. Prior studies have demonstrated that patients with NDMM tend to have more pain and fatigue than those with later-stage disease [26], and a study by Jordan et al. demonstrated that pain and fatigue are the strongest predictors of HRQoL [27]. Treatments that impact these symptoms may therefore have the largest impact on patients’ HRQoL.

Although improvements in the D-VMP group were statistically greater than those in the VMP group on some scores at some time points, between-group differences were largely nonsignificant. This observation needs to be considered in the context of the significant increase in clinical benefits observed with the D-VMP regimen [10]. One possible explanation for the lack of incremental benefit for D-VMP over VMP on HRQoL outcomes may be that this was an on-treatment analysis, in which PRO results are reported for patients remaining on treatment and do not reflect the impact of disease progression resulting in discontinuation of study treatment. A greater proportion of patients in the VMP group compared with the D-VMP group discontinued treatment owing to disease progression (13.3% vs 6.6%) [10]. A second explanation may be the substantial positive impact of bortezomib on HRQoL. The magnitude of symptom improvement observed in the present study is noticeably larger than has been observed in some other studies involving patients with NDMM who were transplant ineligible. For example, the magnitude of the mean changes in GHS, physical functioning, pain and fatigue observed in the D-VMP and VMP groups in the present trial is larger than those observed in the phase III FIRST study of lenalidomide plus low-dose dexamethasone vs melphalan/prednisone/thalidomide [28]. Although cross-trial comparisons need to be interpreted with caution, especially as patient inclusion criteria may differ, these observations suggest that owing to the large improvement in HRQoL with VMP alone and high baseline scores, there was little additional room for improvement in HRQoL upon further addition of daratumumab (i.e. ceiling effect). Especially when it comes to depth of remission, bortezomib-based regimens have consistently reported greater proportions of patients in compete response when compared with immunomodulatory drug–based combinations. In ALCYONE, response assessment was complemented by measurement of MRD rather than by pure International Myeloma Working Group uniform response criteria. In fact, patients who reached MRD-negative status had significantly improved outcomes compared with MRD-positive patients in terms of EORTC QLQ-C30 GHS and pain scores.

Although patients were not randomized by subgroup, and subgroup analyses should be interpreted with caution, results of these analyses were generally supportive of the findings in the overall population. Subgroup analyses also demonstrated symptom improvement with both D-VMP and VMP irrespective of age and functional status. Notably, improvements were observed in patients 75 years of age or older and those with poor overall function, indicating that the addition of daratumumab did not negatively affect HRQoL, even in frail and elderly patients who may have limited treatment options. The improvement in HRQoL in older patients is noteworthy, as elderly patients tend to have greater health impairment, including comorbidities, and so may have a lower likelihood of achieving treatment benefit; and transplant-ineligible patients tend to be older than those who are eligible for transplant [26]. A further subgroup analysis found that improvements in HRQoL were greater for patients achieving the greatest clinical response. This latter observation is consistent with the results of previous studies that have demonstrated an association between improved HRQoL outcomes and depth of clinical response in patients with MM [29, 30].

Other studies have also examined the impact of daratumumab as part of first-line treatment on HRQoL in patients with transplant-eligible and -ineligible MM. In the CASSIOPEIA study, daratumumab in combination with bortezomib, thalidomide, and dexamethasone was associated with significantly greater reductions in pain, less deterioration of cognitive functioning and greater improvements in emotional functioning vs bortezomib, thalidomide and dexamethasone alone in patients with transplant-eligible NDMM [31]. In the MAIA study, the combination of daratumumab with lenalidomide and dexamethasone was associated with faster and sustained improvement in HRQoL measures compared with lenalidomide and dexamethasone alone in patients with transplant-ineligible NDMM [32]. In both the CASSIOPEIA and MAIA studies, improvements in HRQoL were consistent with observed clinical benefit. Our results, the first in a study that includes an alkylator agent, add to these existing data and demonstrate that the combination of D-VMP improves HRQoL, with meaningful improvements in both functional and symptom scales in patients with transplant-ineligible NDMM.

As noted above, the improvements in PROs reported here complement the significant clinical benefits observed with D-VMP vs VMP, including a lower risk of disease progression and higher percentages of patients with MRD negativity. PROs provide the patient perspective on treatment, and use of clinical endpoints and PROs together best reflect the full spectrum of patients’ disease as well as the overall effectiveness of treatment. However, whereas the clinical assessments showed significant improvements with D-VMP vs VMP [10], including significant improvements in overall survival [33], differences in HRQoL between groups were modest and largely nonsignificant. In addition to the two explanations provided above, this disparity could be due to the use of generic PRO instruments in the ALCYONE trial. MM-specific PRO measures with greater sensitivity to changes in HRQoL, symptoms and impacts for comparing two treatments with multiple drugs may have been able to tease out the treatment differences with greater specificity, although the EORTC QLQ-C30 and EQ-5D-5L are validated tools that are widely used to assess HRQoL in patients with cancer.

One of the limitations of the present study is the open-label design, which may lead to biased treatment effects on PROs. As noted above, another limitation is that only on-treatment results are presented, as patients were censored from the analysis when they discontinued treatment, so HRQoL outcomes do not reflect disease progression (and more patients in the VMP group progressed and discontinued treatment). Furthermore, no reasons were documented for missing data, and some consequences of treatment may not have been identified. This study is also limited by the lack of control for the use of pain medication. Nonsteroidal anti-inflammatory drugs are not recommended in patients with MM because of renal toxicity [34], yet 25.7% of patients in the D-VMP group and 14.4% in the VMP group were treated with these agents, and the proportion of patients treated with analgesic, low-dose corticosteroid and anti-inflammatory medications was greater in the D-VMP group than in the VMP group. It is not possible to determine to what extent these medications may have contributed to the decreases in pain observed in the study, although the impact of systemic corticosteroids is likely minimal given the high cumulative dose of prednisone patients received as part of their study treatment. Furthermore, the difference in the proportion of patients treated with these agents while on study treatment may be explained, at least in part, by the fact that more patients remained on treatment in the D-VMP group.

In conclusion, patients with NDMM who were transplant ineligible demonstrated early and continuous improvements in HRQoL, including improvements in function and symptoms following treatment with D-VMP or VMP. Functional status and well-being were maintained in patients who remained in the study for both the D-VMP and VMP treatment groups, and support the clinical efficacy benefits already reported [10]. This analysis highlights the importance of measuring HRQoL and PROs to confirm the benefits of cancer therapy on the day-to-day aspects of patients’ lives, as well as their clinical prognoses.

## Supplementary Information

**Additional file 1: Supplementary Fig. 1** CONSORT patient flow diagram including completion of PRO questionnaires at baseline (ITT population).

**Additional file 2: Supplementary Fig. 2** LS mean change from baseline in EORTC QLQ-C30. **a** – Role functioning. **b** – Emotional functioning. **c** – Cognitive functioning. **d** – Social functioning. **e** – Nausea and vomiting up to 36 months (ITT population).

## Data Availability

The data sharing policy of Janssen Pharmaceutical Companies of Johnson & Johnson is available at https://www.janssen.com/clinical-trials/transparency. As noted on this site, requests for access to the study data can be submitted through Yale Open Data Access (YODA) Project site at http://yoda.yale.edu.

## Abbreviations

ASCT: Autologous stem cell transplant
CI: Confidence interval
D-VMP: Daratumumab plus bortezomib, melphalan and prednisone
ECOG: Eastern cooperative oncology group
EORTC QLQ-C30: European organisation for research and treatment of cancer quality of life
EQ-5D-5L: EuroQol 5-dimensional descriptive system
GHS: Global health status
HR: Hazard ratio
HRQoL: Health-related quality of life
ITT: Intent to treat
LS: Least squares
MID: Minimally important difference
MM: Multiple myeloma
MRD: Minimal residual disease
NDMM: Newly diagnosed multiple myeloma
PRO: Patient-reported outcome
VAS: Visual analog scale
VMP: Bortezomib, melphalan and prednisone
