# Supplementary figures and images for: Health-related quality of life in patients with newly diagnosed multiple myeloma ineligible for stem cell transplantation: results from the randomized phase III ALCYONE trial
Source: BMC Cancer. 2021 Jun 2;21:659. doi: 10.1186/s12885-021-08325-2 (PMC8170980; doi:10.1186/s12885-021-08325-2)

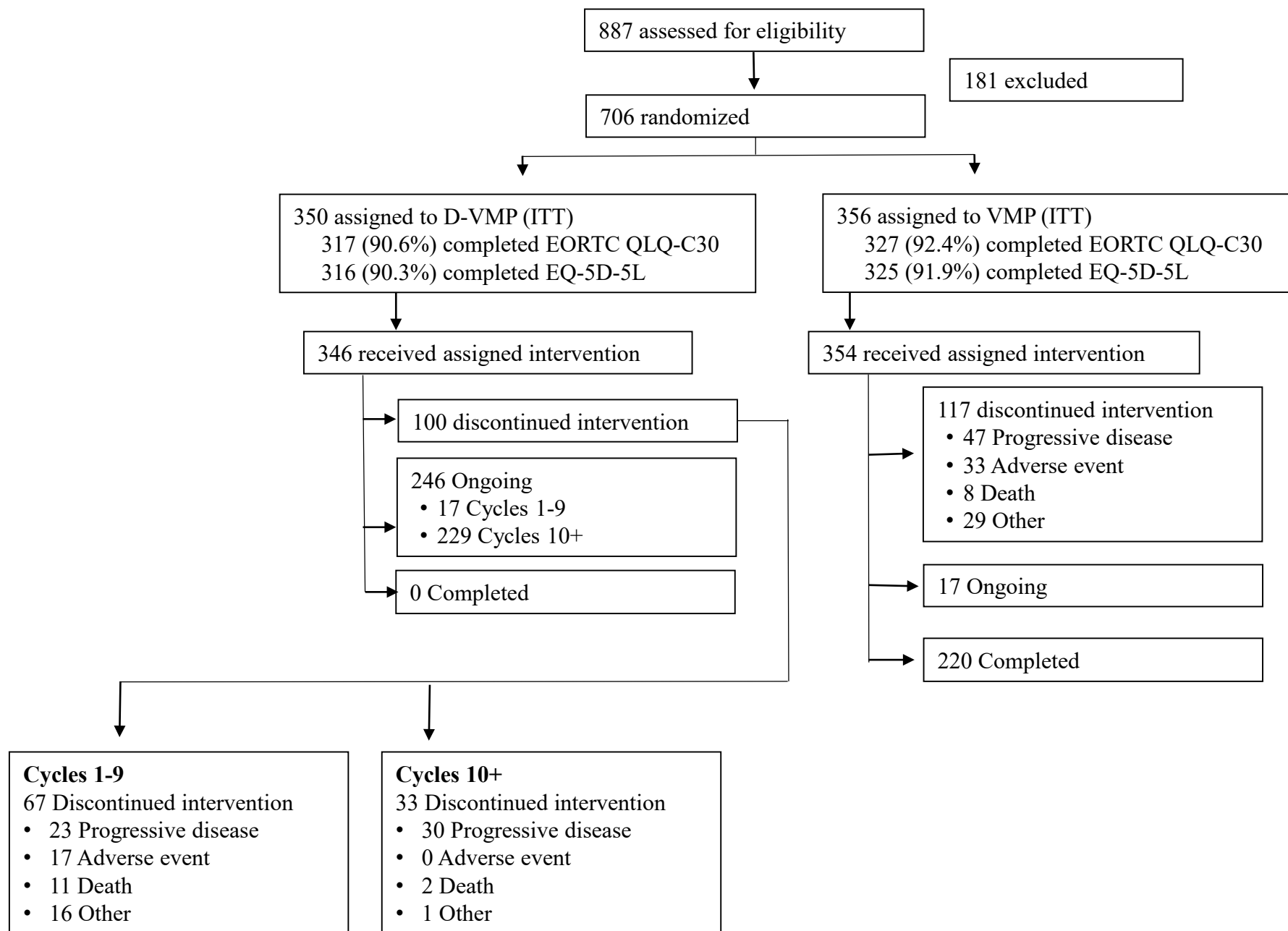

Supplement: Supplementary file 1 — Additional file 1: Supplementary Fig. 1 CONSORT patient flow diagram including completion of PRO questionnaires at baseline (ITT population). [file 12885_2021_8325_MOESM1_ESM.pdf]

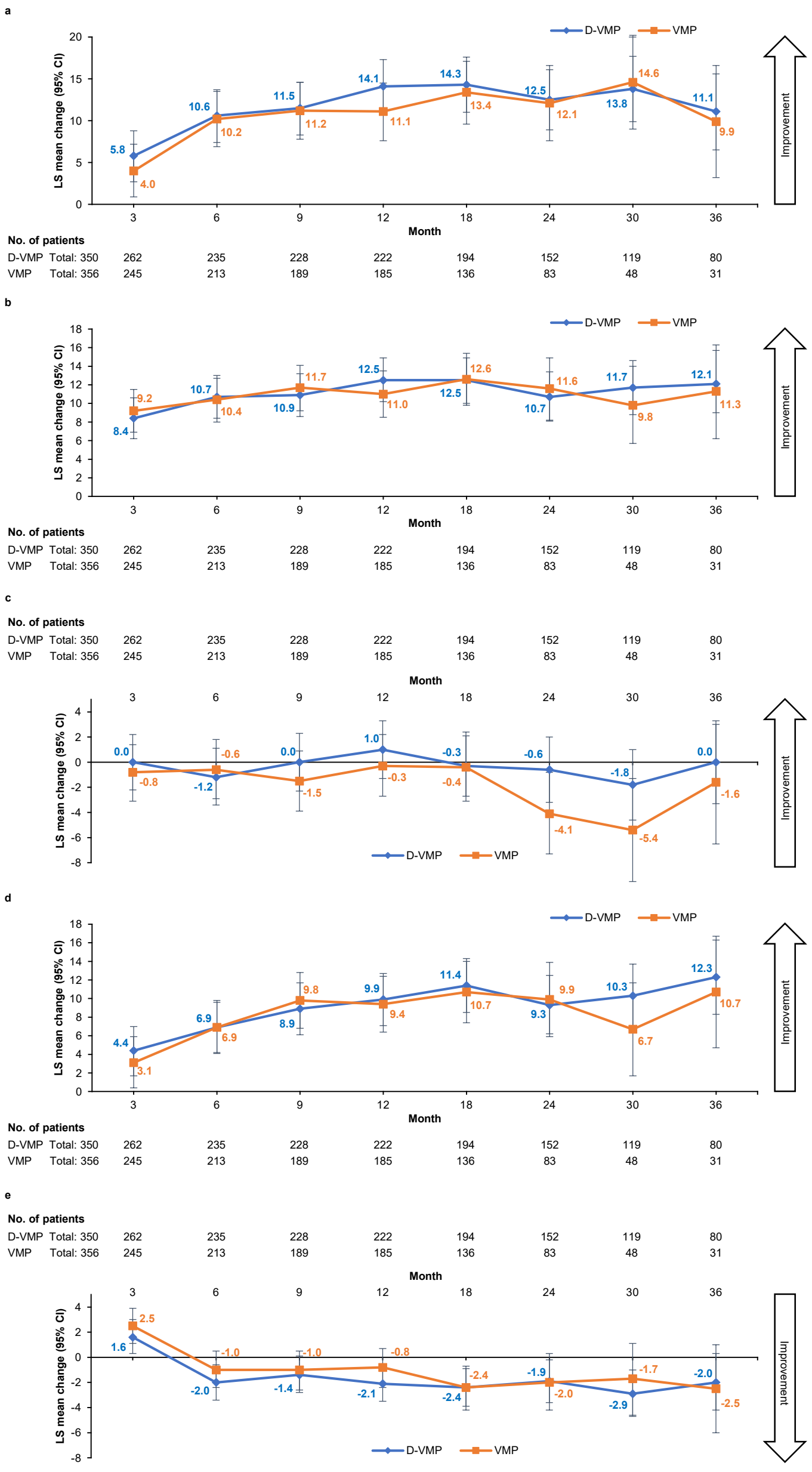

Supplement: Supplementary file 2 — Additional file 2: Supplementary Fig. 2 LS mean change from baseline in EORTC QLQ-C30. a – Role functioning. b – Emotional functioning. c – Cognitive functioning. d – Social functioning. e – Nausea and vomiting up to 36 months (ITT population). [file 12885_2021_8325_MOESM2_ESM.pdf]
